# Anti-VEGF Agents Clearance Through the Aqueous Outflow Pathway in a Rat Model

**DOI:** 10.1167/iovs.66.6.1

**Published:** 2025-06-02

**Authors:** Assaf Ben-Arzi, Itay Spector, Yariv Keshet, Orly Gal-Or, Irit Bahar, Assaf Dotan

**Affiliations:** 1 Department of Ophthalmology, Rabin Medical Center, Petach Tikva, Israel; 2 Faculty of Medicine, Tel Aviv University, Tel Aviv, Israel; 3 Histospeck LTD Labs, Tel-Aviv, Israel

**Keywords:** anti-vascular endothelial growth factor (VEGF), ocular hypertension, brown Norway rat model, aqueous outflow pathway

## Abstract

**Purpose:**

Sustained increase in intraocular pressure (IOP) following intravitreal injection of anti-vascular endothelial growth factor (anti-VEGF) in the treatment of retinal disease has been theoretically attributed to aggregation of anti-VEGF in the iridocorneal angle. However, previous studies by our group showed full clearance of intravitreally injected bevacizumab, aflibercept, and ranibizumab. The objective of this study was to further analyze and compare the clearance of these anti-VEGF agents from the eye after a single injection in a rat model.

**Methods:**

Brown Norway rats received an intravitreal injection of 3 µl anti-VEGF at the standard concentration 3 days following induction of choroidal neovascularization. The eyes were processed at 0, 3, 6, 24, and 48 hours thereafter, and immunofluorescence was evaluated with confocal microscopy and 3D reconstruction analysis. The signal concentration was calculated, and the drug clearance rate was measured. The immunohistochemistry process was validated with negative and positive control groups.

**Results:**

The immunofluorescent signal was positive for all anti-VEGF agents at the trabecular meshwork, Schlemm’s canal, and episcleral veins. Anti-VEGF immunostaining peaked immediately after injection and then decreased gradually to negligible at 48 hours (*P* < 0.05). All three agents demonstrated an identical pattern (*P* > 0.05). The clearance rate of anti-VEGF from the iridocorneal angle ranged between 98.68% and 99.87% at 48 hours.

**Conclusions:**

Bevacizumab, aflibercept, and ranibizumab cleared completely from the iridocorneal angle at 48 hours in the brown Norway rats following a single intravitreal injection and with similar a clearance rate. These findings support our earlier studies refuting the anti-VEGF aggregation hypothesis.

Vascular endothelial growth factor (VEGF) plays an essential role in the formation and growth of new blood vessels. However, overexpression in the eye can lead to pathological neovascularization and increasing vascular permeability, resulting in visual loss.^1^^,^^2^ The introduction 2 decades ago of ant-VEGF agents revolutionized the therapeutic approach to retinal diseases. Studies of intravitreal injection of anti-VEGF reported visual improvement in patients with blinding and irreversible retinal disorders, including exudative age-related macular degeneration, retinal vein occlusion, diabetic retinopathy, and diabetic macular edema.^3^^–^^5^

The three most common anti-VEGF agents currently used in clinical practice are bevacizumab (Avastin; Roche), aflibercept (Eylea; Bayer), and ranibizumab (Lucentis; Novartis). Bevacizumab is a full-length recombinant humanized murine monoclonal antibody (149 kilodalton [kDa]), that binds to and inhibits the biological activity of VEGF-A isoforms. Aflibercept, a recombinant fusion protein (115 kDa), consists of a VEGF-binding receptor segment fused to the fragment crystallizable region of IgG1. Aflibercept inhibits both VEGF-A and VEGF-B isoforms, as well as placental growth factor. Ranibizumab is a recombinant humanized IgG1 kappa monoclonal antibody Fab fragment (48 kDa) that binds to the receptor-binding site of VEGF-A isoforms. A recent fourth agent is faricimab (Vabysmo; Roche), a humanized bispecific immunoglobulin G monoclonal antibody which binds and neutralizes Ang-2 and VEGF-A. Faricimab has been associated with low rates of adverse ocular events such as ocular hypertension.^6^

All intravitreal anti-VEGF agents, including bevacizumab, ranibizumab, and aflibercept, may have adverse effects. One of the most common is sustained elevated intraocular pressure (IOP) which can occur even in patients with no history of ocular hypertension nor glaucoma.^7^^–^^9^ The etiology of sustained elevation IOP after anti-VEGF injection is unknown. Various studies have suggested potential underlying pathophysiologic mechanisms.^10^^–^^20^ According to the mechanical microaggregation hypothesis, the increased IOP is caused by blockage (from protein aggregates of drugs, syringes, and silicone oil from the syringe barrel or rubber stopper) at the trabecular meshwork and Schlemm's canal.^14^^,^^15^ Other possible causes include a direct effect similar to glucocorticoids on extracellular matrix deposition in the trabecular meshwork due to alterations in gene expression; long-term VEGF antagonism that downregulates nitric oxide levels, affecting the outflow dynamics; antagonism of VEGF function as a paracrine regulator of outflow facility; drug-induced trabeculitis; and repeated trauma and/or anti-VEFG-injection-associated IOP spikes, leading damage to the trabecular meshwork.^10^^–^^20^

Previous studies by our group on the same rodent model of brown Norway rat eyes showed the presence of anti-VEGF agents in the aqueous outflow pathway after a single intravitreal injection. The anti-VEGF agents decreased gradually over time and were completely absent by 48 hours.^21^^–^^23^ These findings indicate that anti-VEGF did not accumulate in the iridocorneal angle after a single injection, negating the mechanical microaggregation hypothesis in these conditions. The main objective of the present study was to examine and compare the trends and differences in the clearance from the iridocorneal outflow tracts following a single injection of bevacizumab, aflibercept, and ranibizumab in a brown Norway rat model after exclusion of microaggregation. The brown Norway rat is a well-established model for experimental glaucoma and experimental laser-induced choroidal neovascularization (CNV) sharing similar anatomy to humans and suitability for animal to human translation.

## Methods

### Animal Care and Groups

The study was conducted in brown Norway rats weighing 200 to 300g each (Envigo RMS, Indianapolis, IN, USA). The rats were housed in the Experimental Animal Facility of Felsenstein Research Center. They were maintained under controlled conditions of light (12-hour light-dark cycles with light intensity within the cages ranging from 5 to 30 lux) and temperature (23°C–24°C), with access to food and water ad libitum, in accordance with the Association for Research in Vision and Ophthalmology (ARVO) Statement for the Use of Animals in Ophthalmology and Vision Research. The protocol was approved by the Animal Care and Use Committee of Rabin Medical Center.

The study was designed and performed as described in previous studies.^21^^–^^23^ Twelve rats were used to evaluate each drug included in the study (total = 36 rats). Three groups were defined for each drug: a test group consisting of the right eye of each rat (total = 12 eyes); a positive control group consisting of the left eye of four rats; and a negative control group consisting of the left eye of eight rats.

### Induction of Choroidal Neovascularization

Examination of the aggregation of anti-VEGF agents in the presence of a considerably large amount of VEGF is fundamental to understanding the underlying mechanism. The procedure was based on previous studies suggesting that VEGF has a paracrine effect on the outflow tract, facilitating aqueous clearance from the iridocorneal angle,^24^ and that VEGF concentration in the vitreous cavity increases significantly following laser treatments.^25^ Therefore, CNV was induced in the test groups by indirect laser photocoagulation to create an environment mimicking an increase of VEGF in the eye, as reported previously.^25^

**Figure 1.**
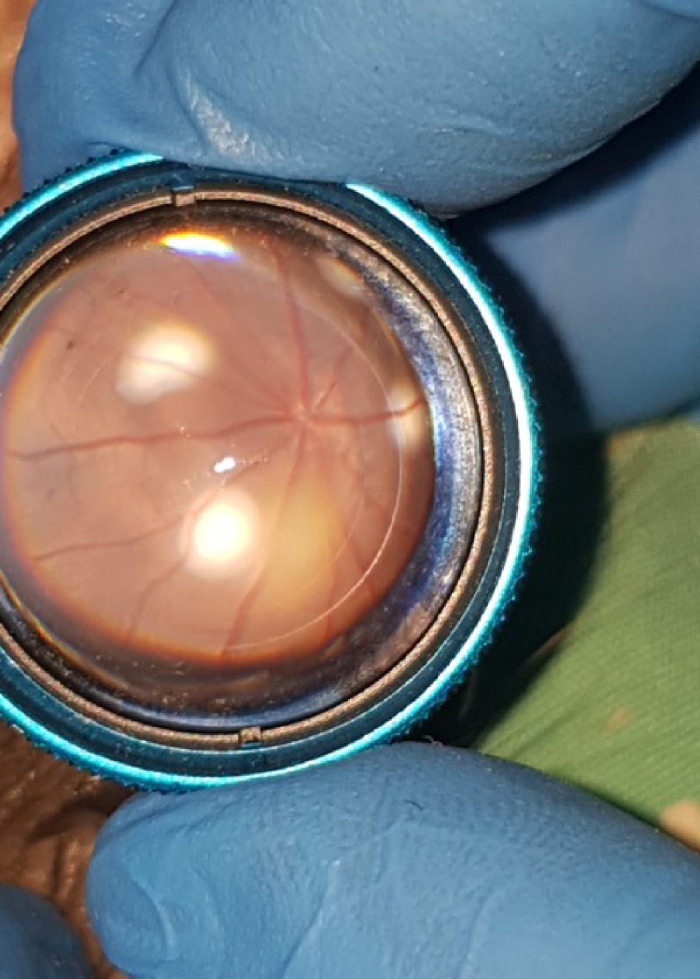
CNV induction. CNV was induced by indirect diode laser photocoagulation in a brown Norway rat eye of the test group. Note the optic nerve with the main vasculature of the retina, arteries, and veins. Laser applications appear as *white-yellow spots*, one in every quadrant around the optic nerve at approximately 2 optic disc diameters.

The rats were placed under general anesthesia by intramuscular administration of ketamine hydrochloride, 50 mg/kg body weight, and xylazine, 5 mg/kg body weight. The pupil was dilated with phenylephrine hydrochloride (0.5%) and tropicamide (0.5%) eye drops supplemented with topical anesthesia (0.5% proparacaine hydrochloride). Laser was applied with a 90-diopter condensing lens at the posterior pole, at the 3, 6, 9, and 12 o'clock positions, at a distance of 1 to 2 optic disc diameters surrounding the optic nerve, until the burn produced a bubble indicating rupture of Bruch's membrane (Fig. 1).

### Intravitreal Injections

Three days after laser photocoagulation, an anti-VEGF agent was injected into the vitreous. The procedure was done with the rats under general anesthesia using a Hamilton bevel-tip 30-gauge needle inserted at a 45-degree angle 1 mm posterior to the limbus. Each eye in the study group and the positive control group received 3 µl of anti-VEGF with a human dose equivalent. The concentration of each agent was determined according to standard practice: bevacizumab = 1.25 mg/0.05 mL; aflibercept = 2 mg/0.05 mL; and ranibizumab = 0.5 mg/0.05 mL.

### Tissue Processing

The rats were euthanized by intracardiac pentobarbital sodium 20% solution at 0, 3, 6, 24, 48, and 72 hours after intravitreal anti-VEGF injection (2 rats in each time point), and the eyes were enucleated, immediately fixated in 4% paraformaldehyde, and embedded in paraffin (formalin-fixed paraffin-embedded [FFPE] tissue) for preparation of 5 µm sections.

### Immunohistochemistry

**Figure 2.**
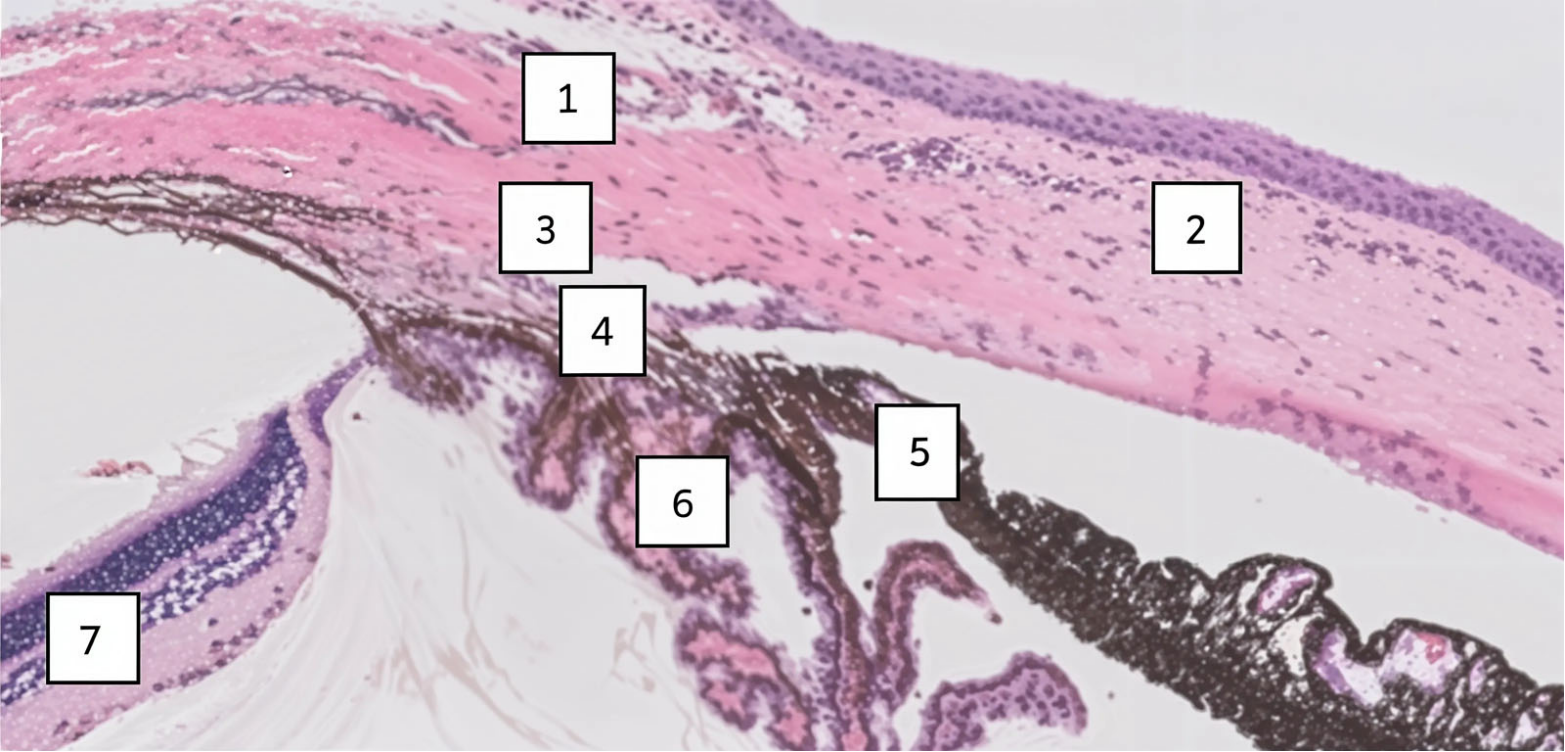
Anatomy and structures of the iridocorneal angle. H&E-stained sections from a brown Norway rat eye showing: (1) episcleral veins; (2) cornea; (3) Schlemm's canal; (4) trabecular meshwork; (5) iris; (6) ciliary body; and (7) retina. Magnification × 2, Zoom 1.

The sections were de-paraffinized and fluorescence immunostained. The process included incubation with the primary antibodies in a humidity chamber for 1 hour at room temperature, afterward, the sections were washed, and incubated with secondary antibodies. Nuclei were detected by 4',6-diamidino-2-phenylindole (DAPI; BLG-422801; Biolegend, San Diego, CA, USA) as a counterstain. Hematoxylin and eosin (H&E) staining was performed in all groups for the purpose of anatomic orientation (Fig. 2).

In the bevacizumab study, mouse anti-rat CD31 was used as a marker for Schlemm’s canal endothelial cells; donkey anti-mouse IgG labeled with Alexa Fluor 568 (#A10037; Molecular Probes, Eugene, OR, USA) served as a secondary antibody for anti-CD31 (red fluorescence). In the aflibercept and ranibizumab studies, immunohistochemistry was performed to detect rat smooth muscle using donkey anti-mouse monoclonal anti-αSMA (#202M-94; Cell Marque, Rocklin, CA, USA) conjugated with Cy2 (green fluorescence): excitation 492 nm, and emission 508 nm.

The primary and secondary antibodies for anti-VEGF agents were as follows**:** bevacizumab – primary antibody: human IgG (709-545-149; Jackson ImmunoResearch, West Grove, PA, USA); secondary antibody: donkey anti-human IgG labeled with Alexa Fluor 488 (Jackson ImmunoResearch); aflibercept – primary antibody: rabbit polyclonal anti-human IgG (269A, 711-165-152; Cell Marque); secondary antibody: donkey anti-rabbit Cy3: excitation 554 nm, emission 568 nm (Jackson ImmunoResearch); ranibizumab – primary antibodies: goat polyclonal anti-human IgG-Fab (A80-114A; Fortis Life Sciences, Waltham, MA, USA) and rabbit polyclonal anti-human IgG (H/L) (STAR195; Bio-Rad, Netiv HaOr, Haifa, Israel); secondary antibodies: donkey anti-goat Cy3, excitation 554 nm, emission 568 nm, and donkey anti-rabbit Cy5: excitation 649 nm, emission 667 nm (Jackson ImmunoResearch). Two different reagents were used for the demonstration of ranibizumab to strengthen and amplify fluorescent signal outcomes due to its biomolecular properties composed of Fab alone. The chosen anti-IgG targets both Fc and Fab; and the anti-Fab targets Fab alone.

### Control Groups

The immunohistochemistry process was validated with negative and positive control groups.

The positive control groups consisted of the left eyes that underwent intravitreal anti-VEGF injection without prior laser photocoagulation for CNV induction. The eyes were immunostained with all primary and secondary antibodies. The rats in the positive control group were euthanized immediately after intravitreal injection of the anti-VEGF agent.

The negative control groups consisted of the left eyes that did not undergo any intervention, either laser photocoagulation or intravitreal injection. The eyes were incubated only with secondary antibodies, and immunostained with DAPI alone.

### Image Acquisition and Analysis

In the bevacizumab study, the staining pattern of the tissue sections was observed under a standard fluorescence microscope and camera settings (Olympus Optical Co., Tokyo, Japan). In the aflibercept and ranibizumab studies, the staining pattern of the tissue sections was observed under a confocal laser scanning microscope (Leica TCS SP 8; Leica Microsystems, Wetzlar, Germany) at magnification × 20, zoom 1 and 2. All photographs of the latter were taken using the same exposures to compare signal intensity between the groups. Three-dimensional (3D) volumetric image analysis was performed with Imaris software (Bitplane, Oxford Instruments, Abingdon, UK) to clearly demonstrate the location of the structures of interest in the immunostained FFPE tissue sections, together with side-view image analysis to precisely demonstrate the signal concentrations (pixels/field).

Signal concentration of anti-VEGFs was calculated as the average number of fluorescent signals from several different sections. The field of signals was made from a whole fluorescent section. In the test group, four to five sections from two different rats were analyzed for every time point. In the negative control group, nine sections from two eyes were included in the calculation; in the positive control group, three to nine sections from three eyes were included.

Brightfield imaging was used in combination with other photography channels in each sample with signals from different antibodies to construct multiple immunohistochemistry signals in the same image.

### Clearance Calculation

Drug clearance in general medicine is defined as the volume of plasma cleared of a drug over a specified period.^26^ In the present study, we have measured the percentage amount of anti-VEGF released over 48 hours as a surrogate for a clearance parameter from the outflow tracts by dividing the “amount of anti-VEGF released” with the “amount of anti-VEGF at time zero” within a constant time frame of 48 hours; the outcome was presented in percentage. The “amount of anti-VEGF agent released” was defined as the “amount of anti-VEGF at time zero” minus the “amount of anti-VEGF at 48 hours.” The analysis of signal concentration (pixels/field) was the average number from four to five whole fluorescent sections from two different rats for every time point.

### Statistical Analysis

The results were visualized using Prism (GraphPad) and Excel (Microsoft Corp.) software. Statistical analysis results were expressed as mean ± standard error. Comparison was performed using the Wilcoxon signed rank test, Tukey’s multiple comparisons test, and *t*-test. Statistical significance was defined as *P* < 0.05. The bar chart was used to visualize the comparison between anti-VEGFs with error bars representing 95% confidence interval (CI).

## Results

During the study, all procedures were completed successfully. There were no adverse events, infections, or premature deaths following a medical procedure. There were no cases of lens trauma or severe vitreous bleeding. No damage was seen throughout the laboratory tissue processing, and no technical problems were encountered, including injuries to the animals during the research period.

### Qualitative Image Analysis

**Figure 3.**
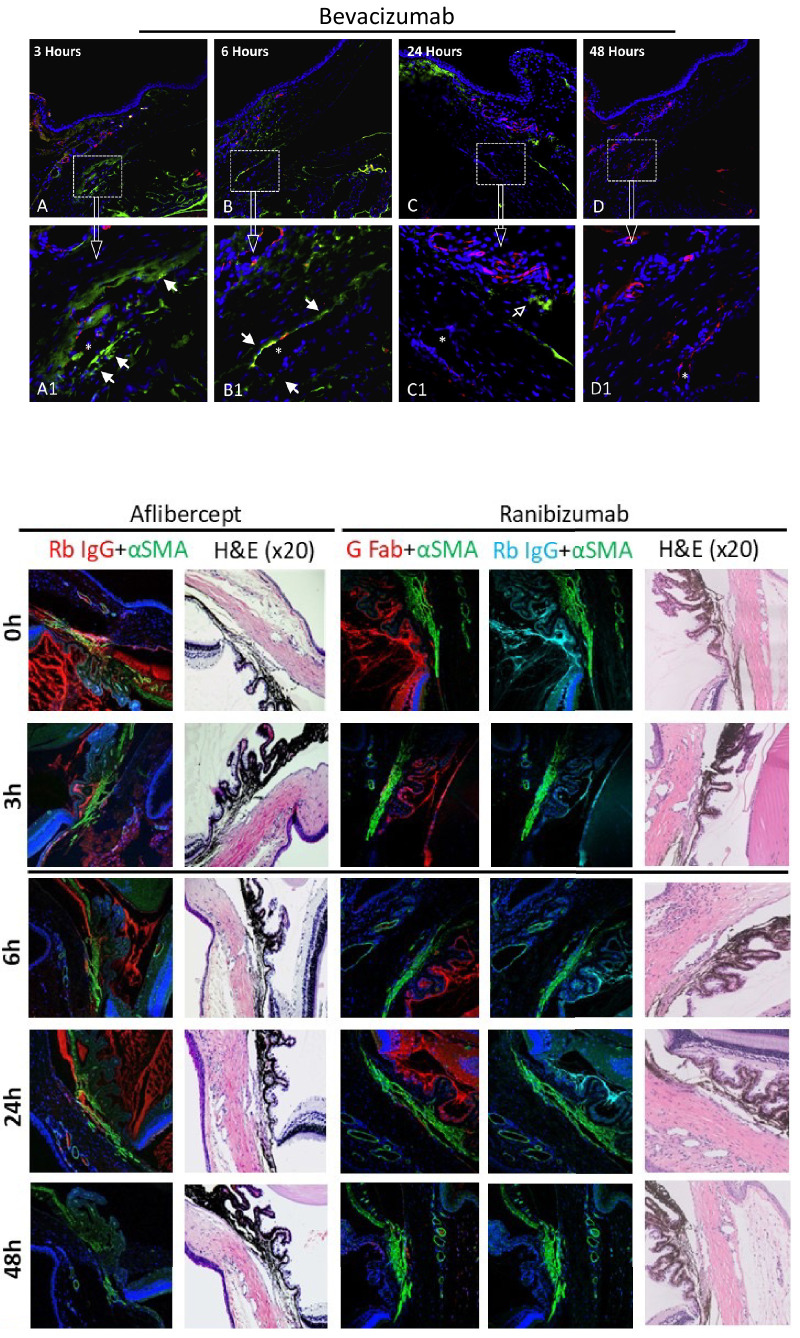
Clearance of anti-VEGF agents. Clearance was evaluated by immunostaining signals in the test groups throughout the timeline of the studies. Immunoreactivity gradually decreased until the immunofluorescent signal was completely negligible after 48 hours. The bevacizumab sections are presented in horizontal ascending order from 3 hours to 48 hours; the *top row* is at low magnification, and the *bottom row* is at high magnification (× 100). Mouse anti-rat CD31 (*red*) and DAPI (*blue*) were used as markers for anatomic orientation and detection of Schlemm canal (SC) endothelial cells. Alexa Fluor 488 conjugated donkey anti-human IgG antibody (*green*) was used to detect bevacizumab immunoreactivity. The aflibercept and ranibizumab sections are presented in vertical ascending order from time zero to 48 hours. Mouse monoclonal anti-αSMA (*green*) and DAPI (*blue*) were used for anatomic orientation. Adjacent to the immunostained sections are corresponding sections stained with H&E to assess tissue morphology. Aflibercept was detected by rabbit polyclonal anti-human IgG (269A-14 red, “Rb IgG” or “IgG”) and donkey anti-rabbit Cy3: excitation = 554 nm and emission = 568 nm. Ranibizumab was detected by goat polyclonal anti-human IgG-Fab (A80-114A, red, “G Fab”) and donkey anti-goat Cy3: excitation = 554 nm and emission = 568 nm; rabbit polyclonal anti-human IgG (H/L; #STAR195, pale-blue, “Rb IgG” or “IgG”) and donkey anti-rabbit Cy5: excitation = 649 nm and emission = 667 nm. pH6, high conc., magnification × 20, Zoom 1.

The anatomy landmarks of the iridocorneal angle were identified in all eyes with H&E stain (see Fig. 2). Anti-VEGF molecules were observed in the test groups eyes (one test group for each drug) within the iridocorneal angle and the adjacent structures in the anterior chamber at time 0, 3 hours, 6 hours, and 24 hours after injection. The immunofluorescent signal was negligible at 48 hours, confirming complete clearance of the agents and eliminating the need for presenting further analysis at 72 hours (Fig. 3).

**Figure 4.**
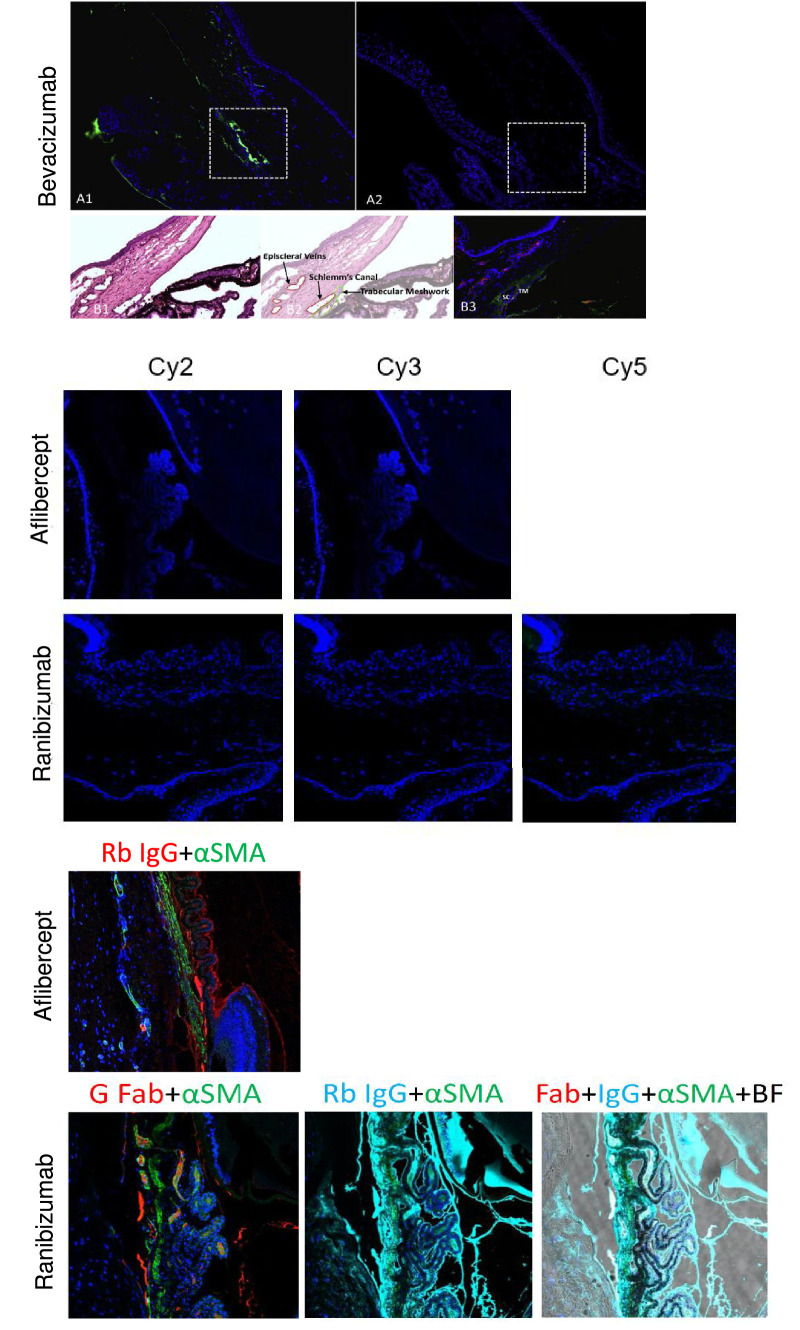
Negative and positive control groups. Negative control samples were processed with secondary antibodies alone. As expected, no immunofluorescent signal was seen. The only fluorescent staining is of DAPI blue, which represents nuclei in the tissue. Bevacizumab (A2), aflibercept, and ranibizumab are presented in the middle. Magnification × 20, Zoom 1. Positive control samples were processed with all primary and secondary antibodies. Magnification × 20, Zoom 1. A fluorescent signal is seen from all types of antibodies. Bevacizumab (A1, B3) detection in *green* (anti-human IgG antibody) and endothelial cells in *red* (anti CD31); corresponding H&E (B1) areas with illustration (B2) of trabecular meshwork (TM), Schlemm’s canal (SC) and episcleral veins in the rat’s aqueous drainage tract. Aflibercept and ranibizumab: smooth muscle in *green* (mouse monoclonal anti-αSMA, abbreviated αSMA), aflibercept in *red* (rabbit polyclonal anti-human IgG, abbreviated Rb); ranibizumab in *red* (goat polyclonal anti-human IgG-Fab, abbreviated G Fab), and *pale blue* (rabbit polyclonal anti-human IgG [H/L], “Rb IgG”). BF = brightfield.

In the negative control group (no laser photocoagulation, no intravitreal anti-VEGF, secondary antibodies only, and DAPI immunostaining), no fluorescent signal was seen on incubation with the secondary antibodies other than blue from DAPI, representing the distribution of nuclei in the tissues (Fig. 4).

In the positive control group (intravitreal anti-VEGF, no laser photocoagulation, and immunostaining with all primary and secondary antibodies), photographs taken under immunofluorescent microscopy demonstrated the iridocorneal structures and anti-VEGFs molecules (see Fig. 4).

**Figure 5.**
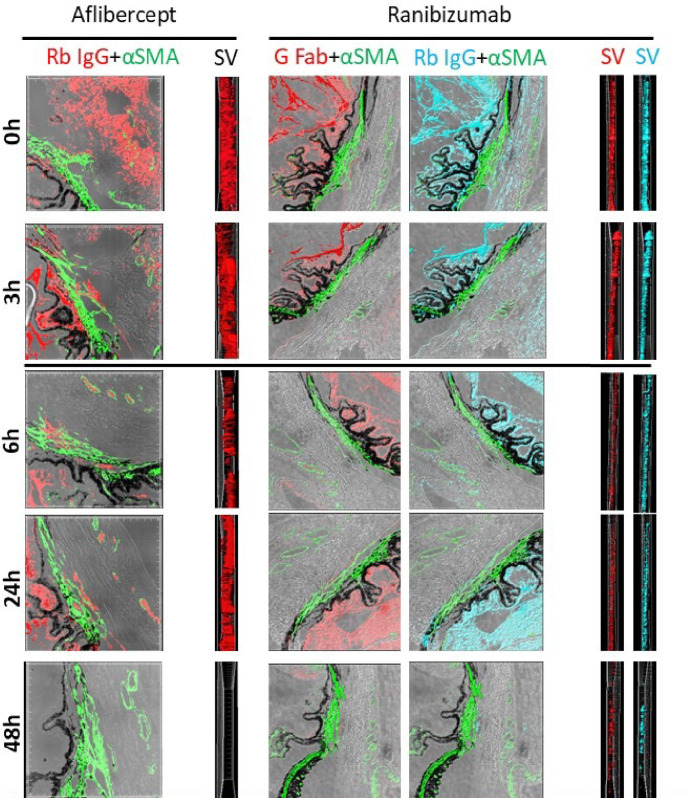
Three-dimensional volumetric reconstruction. Images of aflibercept and ranibizumab in brightfield, with adjacent side views (SVs) to gain depth perspective of the immunofluorescent signal which gradually diminished over time, from 0 to 48 hours. By 48 hours, the signal is negligible. Immunostaining: smooth muscle in *green* (mouse monoclonal anti-αSMA, “αSMA”), aflibercept in *red* (rabbit polyclonal anti-human IgG [269A-14], “Rb IgG”), ranibizumab in *red* (goat polyclonal anti-human IgG-Fab [A80-114A], “G Fab”) and in *pale blue* (rabbit polyclonal anti-human IgG [H/L] (#STAR195, “Rb IgG”); SV in *black* corresponds to aflibercept “Rb IgG”; SV in *red* corresponds to ranibizumab “G Fab” antibody; SV in *pale blue* corresponds to ranibizumab “Rb IgG” antibody. pH6; high conc, magnification × 20, Zoom 1.

The confocal microscopy results were supported by 3D volumetric image reconstruction of brightfield images used for depth assessment of the immunoreactivity signals. Side views of the 3D reconstruction photographs were obtained, which, owing to their depth, facilitated qualitative assessment of signal concentration along the different time points after anti-VEGF intravitreal injection (Fig. 5).

### Quantitative Signal Concentration Analysis

**Figure 6.**
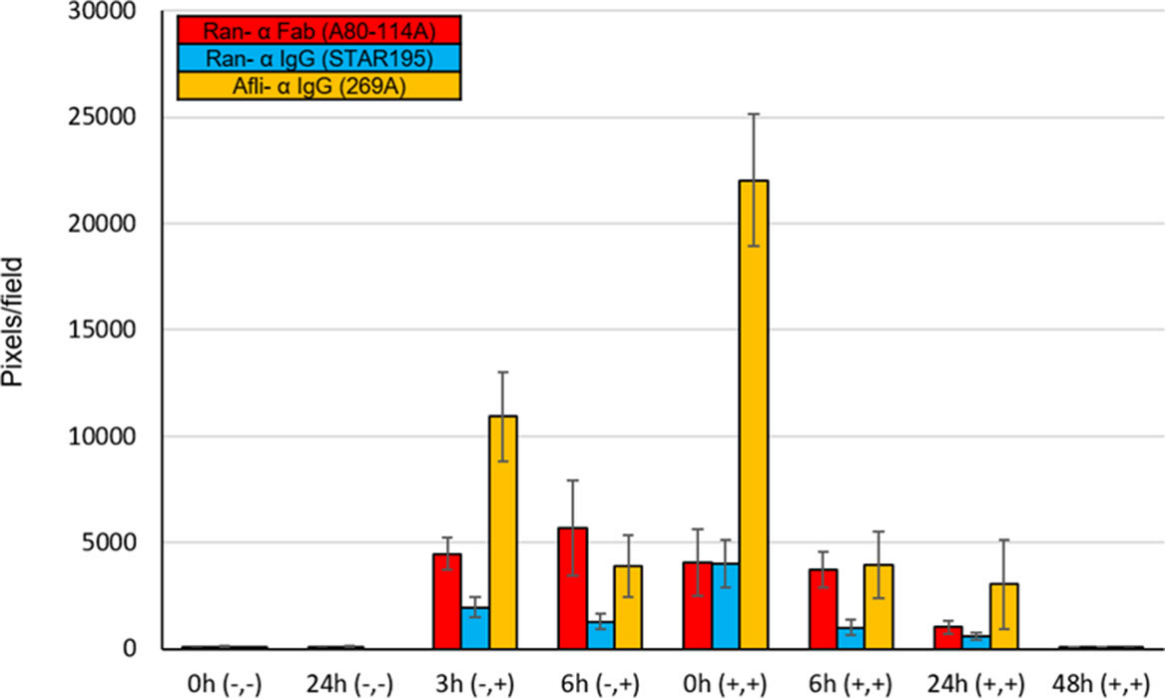
Quantitative analysis. Ranibizumab (Ran- α Fab [A80-114A]) in *red* and (Ran- α IgG [STAR195]) in *blue*; aflibercept (Afli- α IgG [269A]) in *yellow*. Average signal concentration is presented along the Y axis; the value was calculated by pixels/field from three to nine different sections. Columns on the X axis are divided by time: 0 hours (−, −) and 24 hours (−, −) which represents the negative controls; 3 hours (−, +) and 6 hours (−, +) which represents the positive controls; 0 hours (+, +), 6 hours (+, +), 24 hours (+, +), and 48 hours (+, +) represent the test groups. Signal concentration gradually reduces throughout the timeline. At 48 hours, the signals were negligible with no difference among the antibodies and the negative control groups (*P* > 0.5). The signal of aflibercept anti-IgG signal was significantly higher at 0 hours and 3 hours than the signal of ranibizumab anti-Fab and ranibizumab anti-IgG (*P* < 0.01). Error bars represent 95% CI.

The average fluorescent signal strength (pixel/field) is graphically described in a clustered column chart in Figure 6. At time zero, the signal per field was 4069.89 ± 1549.82 pixels/field for ranibizumab anti-Fab, 3991.89 ± 1112.12 pixels/field for ranibizumab anti-IgG, and 22,029.00 ± 3121.98 pixels/field for aflibercept anti-IgG. The aflibercept anti-IgG signal was significantly higher at time zero and at 3 hours than the signal of ranibizumab anti-Fab (*P* < 0.0001, *P* = 0.0085, respectively) and ranibizumab anti-IgG (*P* < 0.0001, *P* = 0.0025, respectively). Signal strength gradually decreased over time for all anti-VEGF antibodies (*P* < 0.05), and at 48 hours, it was negligible for all (< 55 pixels/field): 50.78 ± 23.96 pixels/field for ranibizumab anti-Fab, 52.89 ± 13.97 pixels/field for ranibizumab anti-IgG, and 29.25 ± 11.50 pixels/field for aflibercept anti-IgG; with no significant statistical difference among the fluorescence signal from the antibodies (*P* > 0.5). The percentage amount of anti-VEGF released over 48 hours (see the Methods section) was calculated 98.75% for ranibizumab anti-Fab, 98.67% for ranibizumab anti-IgG, and 99.86% for aflibercept anti-IgG, with no significant statistical difference (*P* > 0.09). Unfortunately, owing to technical issues during imaging acquisition and maintenance, bevacizumab signal clearance could not be reliably evaluated and quantified. Nevertheless, as noted above, on observable qualitative analysis, bevacizumab clearance was seen to be complete at 48 hours (see Fig. 3) and thus could be descriptively compared with the other agents.

### Positive Controls Versus Test Groups

We evaluated and compared the signal concentration for each anti-VEGF agent between the test groups, which underwent laser photocoagulation followed by intravitreal anti-VEGF injection, and the positive control groups which underwent only intravitreal injection. The analysis was performed for every anti-VEGF agent alone. The aflibercept positive control groups (10,912 and 3905 pixels/field) were statistically significantly lower than those of the test group of aflibercept anti-IgG at time zero (22,029 pixels/field; *P* = 0.003 and *P* = 0.0003). The ranibizumab positive control groups of anti-IgG (1950 and 1282 pixels/field) were also statistically significantly lower than the ranibizumab anti-IgG test group at time zero (3991 pixels/field; *P* = 0.043 and *P* = 0.0016); However, the ranibizumab anti-Fab positive control groups (4471 and 5666 pixels/field) were higher than the ranibizumab anti-Fab test group at time zero (4069 pixels/field, *P* > 0.9), although not statistically significant. There was no statistically significant difference at each time point between ranibizumab anti-IgG and anti-Fab.

## Discussion

In this study, we analyzed and compared the estimated percentage of intravitreally anti-VEGF released from the iridocorneal outflow tracts as a surrogate for a clearance parameter in eyes of brown Norway rats. Vitreous clearance may involve both posterior and anterior pathways. Small, lipophilic drugs with a short half-life are cleared by the posterior pathway via paracellular or transcellular routes crossing the retina and arriving at the choroidal blood flow. Large, hydrophilic drugs with a long half-life exit through the anterior route by diffusion and aqueous turnover from the trabecular or uveoscleral outflow.^27^

The large molecular weight anti-VEGF agents investigated here, bevacizumab, aflibercept, and ranibizumab, are the most common anti-VEGF agents used in clinical practice today. All three are associated with sustained intraocular hypertension.^7^^–^^11^ Based on our results; no aggregation occurred with any of the agents following a single intravitreal injection. Qualitative and quantitative analysis using antibodies against aflibercept and ranibizumab fundamentally supported this finding, as did qualitative measures alone using antibodies against bevacizumab. The calculated clearance rates of ranibizumab and aflibercept were remarkably high, as expected (98.7% and 99.8% in 48 hours), with no significant between-group difference. These findings are in line with previous studies from our center using a similar design,^21^^–^^23^ and refute the hypothesis that mechanical obstruction of the trabecular meshwork and Schlemm’s canal accounts for the persistent hypertension that may follow even a single anti-VEGF injection. Yet, it is important to emphasize that the relevancy of our results in these studies which were held on rodent models are inferred as equivalent to outcomes in human solely based on anatomical similarity and estimation and not on definitive measurements in humans, thus prediction to clinical settings should be taken with limited liability and further studies are warranted.

We adopted the method of Kim et al.^25^ for CNV induction in brown Norway rats. It is also similar to the method described by Edelman and Castro.^28^ Kim et al.^25^ confirmed the presence of CNV with fluorescein angiography and by studying VEGF levels in the vitreous. Applying the exact methodology in the present study eliminated the need to repeat the preliminary experiments and allowed us to focus straightaway on the predetermined purpose.

Following CNV induction via laser photocoagulation, there is a significant increase in the concentration of VEGF.^25^ VEGF is suggested to be a paracrine regulator of conventional outflow facility.^24^ In this research, VEGF was not measured, nonetheless, we used the amount of anti-VEGF at time zero (pixel/area) as a surrogate of estimating the effect of high VEGF levels on outflow facility. Our analysis achieved ambiguous results when we compared the test groups’ signal concentration at time zero with that of the positive control groups, which differ only by undergoing laser photocoagulation. Thus, although with no direct measurements of intraocular VEGF concentration and by using a rodent model, in this study, laser induced CNV provided as a surrogate for upregulation of intraocular VEGF had minimal facilitating effect on outflow. In addition, anti-VEGF was proposed to have an antagonistic action on outflow facility.^29^ Our results did not support this assertion due to inconsistency of results regarding aflibercept and ranibizumab, whereas bevacizumab caused technical hurdles that were insurmountable to support this premise. The intravitreal injection procedure was done technically the same to all eyes in the test group and the positive control group and with the same dose of 3 µl and by the same physician, therefore, not explaining the inconsistency in outcomes. The inconsistency in our findings raises questions regarding the function of VEGF as a potent and significant regulator of outflow tract. The different outcomes with IgG and Fab may be explained by several factors: the different antibody molecular structure and antibody binding affinity and avidity, for example, Fab is monovalent whereas IgG is bivalent; outflow facility is more negatively affected by the complex of antigen-antibody (VEGF-anti-VEGF) than anti-VEGF alone; goat polyclonal anti-human IgG-Fab (A80-114A) binding affinity to ranibizumab is higher when VEGF concentration is low; high tissue section variability in the presence of anti-VEGF agents; or due to other minimal technical issues. Further research regarding the effect of the different anti-VEGFs on outflow facility are required to resolve this issue, and the use of anti-IgG and anti-Fab agents may need to be further interrogated to see whether these were optimized for such studies due to these inconsistencies shown in the study.

Bevacizumab, aflibercept, and ranibizumab were reported to be associated with a high rate of increased IOP.^7^^–^^11^^,^^30^^–^^32^ The mean IOP spike following intravitreal injection of bevacizumab was 34.8 ± 10.4 millimeters of mercury (mm Hg), and of ranibizumab, 37.7 ± 8.3 mm Hg.^7^ One study demonstrated a sustained IOP increase of at least 5 mm Hg at 96 weeks for aflibercept and ranibizumab.^32^ In a Cochrane database review, increased IOP after intravitreal injections was one of the most frequently reported serious ocular adverse events.^31^ IOP dynamics have been examined previously in a rat model in other published reports.^33^^–^^36^ The brown Norway rat was chosen as the rodent model for this research above other animal models due to its use in previous relevant studies,^21^^–^^23^^,^^25^ having a pigmentary retina for laser induced CNV, shares similar anatomy for IOP dynamics, and is more accessible due to being a small mammal species. Yet, there are still some limitations for extrapolation of rodent results to humans.

In summary, we evaluated and compared the clearance of anti-VEGFs from the iridocorneal angle in eyes of brown Norway rats after a single intravitreal injection. Our finding of a 99% clearance rate at 48 hours after a single intravitreal injection of bevacizumab, aflibercept, and ranibizumab indicates that there was no mechanical obstruction of the outflow tracts in each of the three anti-VEGF agents which were cleared completely at a similar clearance rate.

### Limitations

This study has several limitations. We are aware that the sample assessed for each VEGF agent was small, but we sought to reduce the number of animals used for research to a necessary minimum. As described earlier, there are limitations regarding rodent to human translation, and limits of the rodent model compared with other species or preclinical models with closer translation. Only four to five sections were chosen per analysis of fluorescent signal strength for technical and practical causes. The study focused on the effects of a single intravitreal injection of anti-VEGF, so the results do not preclude a possible risk of multiple injections. Finally, higher magnification microscopy might have been useful to fully evaluate micro-structural changes in the trabecular meshwork, the extracellular matrix and Schlemm’s canal caused by microparticle aggregation and obstruction.

## Conclusions

This study demonstrated that bevacizumab, aflibercept, and ranibizumab do not aggregate in the iridocorneal angle structures following a single intravitreal injection in a rat model of CNV. All three anti-VEGF agents cleared completely through the outflow tracts at 48 hours after injection. The clearance rate was 99%, with no significant difference among the agents. Further studies are warranted to evaluate the mechanism underlying sustained IOP following intravitreal injections of anti-VEGF.
